# *N*,*N*′-Bis(2-ammonio­benz­yl)ethane-1,2-diammonium–nitrate–perchlorate (1/1.5/2.5)

**DOI:** 10.1107/S160053680904519X

**Published:** 2009-11-04

**Authors:** Luis Angel Garza Rodríguez, Sylvain Bernès, Blanca Nájera Martínez, Perla Elizondo Martínez, Nancy Pérez Rodríguez

**Affiliations:** a Laboratorio de Química Industrial, CELAES, Facultad de Ciencias Químicas, UANL, Pedro de Alba S/N, 66451 San Nicolás de los Garza, NL, Mexico; b DEP Facultad de Ciencias Químicas, UANL, Guerrero y Progreso S/N, Col. Treviño, 64570 Monterrey, NL, Mexico

## Abstract

The title compound, C_16_H_26_N_4_^4+^·2.5ClO_4_^−^·1.5NO_3_^−^, is an organic salt in which the cation is a fully protonated tetra­mine. The cation lies on an inversion center and, as a consequence, both benzene rings are parallel. The central chain is found in an all-*trans* arrangement, a conformation different from that observed in the crystal structure of the non-protonated mol­ecule. The charges are balanced by a mixture of nitrate and perchlorate ions. One site is occupied by an ordered perchlorate ion, while the other contains both nitrate and perchlorate ions, with occupancies of 0.75 and 0.25, respectively. In the crystal, the NH_2_^+^ groups of the cation form N—H⋯O hydrogen bonds with the anions. The NH_3_^+^ groups also behave as donor groups, allowing the building of chains along [100], alternating cations and disordered anions being connected *via* N—H⋯O hydrogen bonds.

## Related literature

For the structure of the free tetra­mine, see: Rodríguez de Barbarín *et al.* (2007 ▶). For the use of polyaza ligands for depolymerization of poly(ethyl­ene terephthalate), see: Carta *et al.* (2003 ▶); Parra *et al.* (2004 ▶); Pohorely *et al.* (2006 ▶).
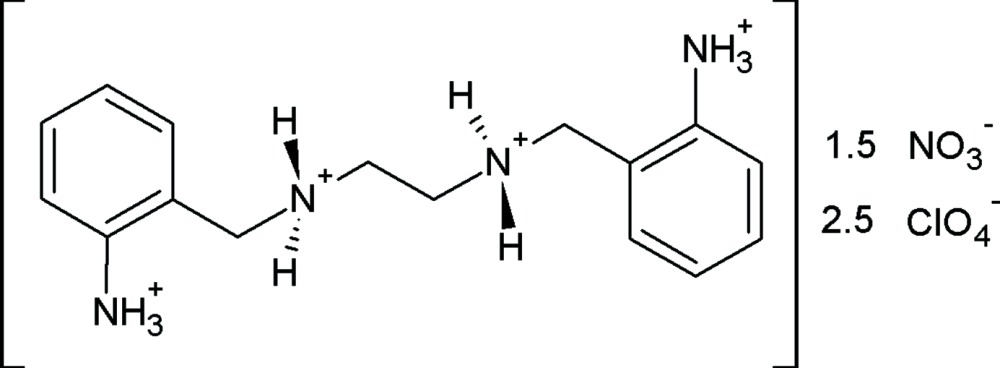


## Experimental

### 

#### Crystal data

C_16_H_26_N_4_^4+^·2.5ClO_4_^−^·1.5NO_3_^−^
*M* *_r_* = 616.05
Monoclinic, 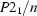

*a* = 8.427 (3) Å
*b* = 12.637 (3) Å
*c* = 11.834 (3) Å
β = 106.97 (2)°
*V* = 1205.4 (6) Å^3^
*Z* = 2
Mo *K*α radiation
μ = 0.41 mm^−1^
*T* = 298 K
0.6 × 0.4 × 0.4 mm

#### Data collection

Siemens P4 diffractometer
Absorption correction: none
6392 measured reflections
2125 independent reflections
1757 reflections with *I* > 2σ(*I*)
*R*_int_ = 0.057
3 standard reflections every 97 reflections intensity decay: <1%

#### Refinement

*R*[*F* ^2^ > 2σ(*F* ^2^)] = 0.050
*wR*(*F* ^2^) = 0.119
*S* = 1.14
2125 reflections
218 parameters
8 restraints
H-atom parameters constrained
Δρ_max_ = 0.25 e Å^−3^
Δρ_min_ = −0.27 e Å^−3^

### 

Data collection: *XSCANS* (Siemens, 1996 ▶); cell refinement: *XSCANS*; data reduction: *XSCANS*; program(s) used to solve structure: *SHELXTL-Plus* (Sheldrick, 2008 ▶); program(s) used to refine structure: *SHELXTL-Plus*; molecular graphics: *SHELXTL-Plus* and *Mercury* (Macrae *et al.*, 2006 ▶); software used to prepare material for publication: *SHELXTL-Plus*.

## Supplementary Material

Crystal structure: contains datablocks I, global. DOI: 10.1107/S160053680904519X/vm2008sup1.cif

Structure factors: contains datablocks I. DOI: 10.1107/S160053680904519X/vm2008Isup2.hkl

Additional supplementary materials:  crystallographic information; 3D view; checkCIF report

## Figures and Tables

**Table 1:** Hydrogen-bond geometry (Å, °)

*D*—H⋯*A*	*D*—H	H⋯*A*	*D*⋯*A*	*D*—H⋯*A*
N9—H9*A*⋯O2	0.90	2.05	2.872 (4)	151
N9—H9*B*⋯O7	0.90	2.02	2.89 (5)	163
N9—H9*B*⋯O13	0.90	1.98	2.836 (13)	157
N1—H1*A*⋯O5^i^	0.89	2.04	2.91 (3)	165
N1—H1*A*⋯O12^i^	0.89	2.05	2.920 (13)	164
N1—H1*B*⋯O8^ii^	0.89	1.90	2.78 (2)	170
N1—H1*B*⋯O14^ii^	0.89	2.14	3.026 (11)	174
N1—H1*C*⋯O1^iii^	0.89	2.39	3.207 (4)	153

Symmetry codes: (i) 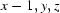; (ii) 
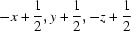
; (iii) 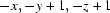.

**Fig. 1.**
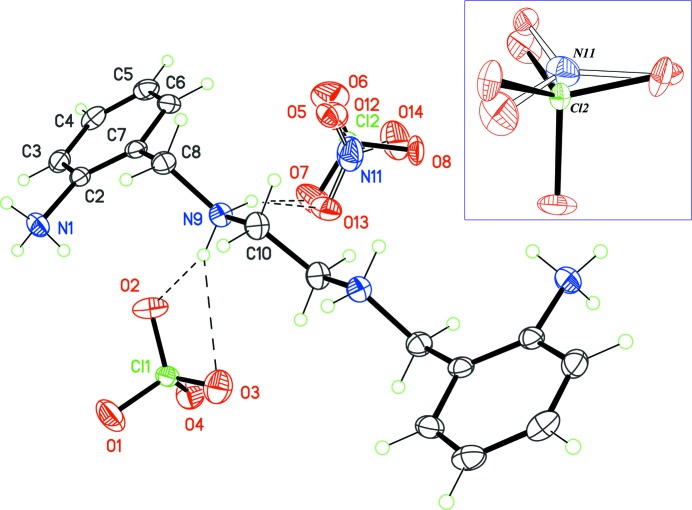
The structure of the title compound, with displacement ellipsoids at the 30% probability level. Dashed lines represent hydrogen bonds in the asymmetric unit. The inset shows the anionic site with two disordered anions: nitrate N11 (occupancy = 3/4) and perchlorate Cl2 (occupancy = 1/4).

**Fig. 2.**
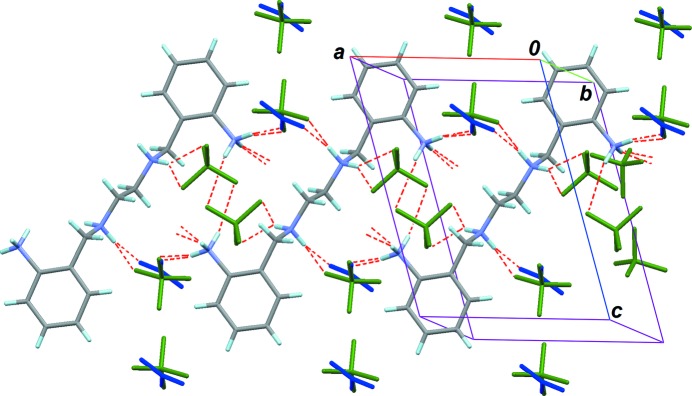
A part of the crystal structure of the title compound, with hydrogen bonds represented with dashed lines. Green anions are perchlorate and blue anions are nitrate. For the sake of clarity, some perchlorate ions have been omitted (hanging contacts). The crystal is viewed almost along [010].

## supplementary crystallographic information

### Comment

Poly(ethylene terephthalate) (PET) is a thermoplastic material, which has been increasingly used in the industry during the last decades. Its low degradability makes this material highly contaminant to the environment (Carta *et al.*, 2003). Currently, many efforts are devoted to reduce the amount of waste PET that reaches the landfillings. Some processes are able to recycle PET into highly valued carbon materials (Parra *et al.*, 2004), used to generate heat and electricity (Pohorely *et al.*, 2006). An interesting approach known as 'chemical recycling' is based on the depolymerization of PET through solvolytic chain cleavage. Five processes have been probed, with different depolymerizing agents: methanolysis, glycolysis, hydrolysis, aminolysis and ammonolysis. Several reactions used catalyst, among them zinc compounds.

Our group is involved in the search for reactions relevant to the chemical degradation of PET, using acyclic polyaza zinc complexes Zn*LX*_2_ (*X* = ClO_4_^-^, NO_3_^-^; *L* = polyaza ligand), which gave excellent results. The title salt appeared during an attempt to prepare a bimetallic catalyst. Previous works showed that complex Cu*L*[CuCl_4_] can be obtained as the product of the transmetallation of Mn*L*(NO_3_)_2_ with Cu(ClO_4_)_2_.6H_2_O in ethanol, with an excess of aqueous HCl. The same transmetallation procedure, using Zn(ClO_4_)_2_.6H_2_O in acidic ethanol afforded pale yellow crystals as a subproduct, which were identified as the title salt by X-ray diffraction.

The salt is composed of a tetracation and a mixture of nitrate and perchlorate anions (Fig. 1). The presence of both anions in the material was confirmed by IR spectroscopy, as spectra include characteristic vibrations for these ions. The cation is fully protonated and is placed on an inversion center. As a consequence, benzene rings are parallel by symmetry. The 6-membered chain linking the benzene fragments is found in the all-*trans* extended conformation, contrasting with the *trans*-*gauche*-*trans* conformation observed in the solid-sate for the non-protonated tetramine (Rodríguez de Barbarín *et al.*, 2007). Anions are found in two sites. One site is occupied by a non disordered perchlorate, Cl1. The other site contains a mixture of nitrate (N11) and perchlorate (Cl2), with occupancies 3/4 and 1/4, respectively (Fig. 1, inset). Because they are involved in hydrogen bonds, anions do not present orientational disorder.

NH_2_^+^ and NH_3_^+^ groups have different functions regarding hydrogen bonding in the crystal. NH_2_^+^ donors groups are connected to anions within the asymmetric unit, forming N—H···O hydrogen bonds (Fig. 1). NH_3_^+^ groups also serve as donors for N—H···O hydrogen bonds, connecting symmetry related cations *via* anions, to form a one-dimensional supramolecular structure where cations and anions alternate in the [100] direction (Fig. 2).

### Experimental

A 25 ml flask was charged with Mn*L*(NO_3_)_2_ [279 mg; *L* is the free tetramine corresponding to the title cation (Rodríguez de Barbarín *et al.*, 2007)] and ethanol (9 ml) and the mixture was stirred for 5 min., giving a white suspension. Salt Zn(ClO_4_)_2_.6H_2_O was added (919 mg) and the reaction further stirred for 3 min., affording a quite clear solution. Immediately, 200 mg of concentrated HCl was added, and the mixture turned to a translucent light yellow solution, which was cooled for 4 d. Light yellow-green crystals were formed over this period, which were isolated and washed with cold ethanol, diethyl ether and finally air dried. Yield 68% (26.2 mg), m.p. 160 °C (dec.). IR (KBr, cm^-1^) ν(CH_2_) 2942, 2787, ν(NO_3_) 1383, ν(ClO_4_) 1084, 940.

### Refinement

All H atoms were placed in idealized positions, with bond lengths fixed to 0.97 (methylene CH_2_), 0.93 (aromatic), 0.90 (NH_2_^+^) and 0.89 Å (NH_3_^+^). Isotropic displacement parameters for H atoms were calculated from displacements of parent atoms. Site occupation factors for nitrate N11 and perchlorate Cl2 anions (Fig. 1, inset) were first roughly refined and finally fixed to 3/4 and 1/4 in order to match the charges balance. The geometry for the nitrate ion was restrained to be flat and N—O bond lengths were restrained to 1.23 (1) Å. For the perchlorate, Cl—O bond lengths were restrained to 1.41 (1) Å.

### Crystal data

**Table:**

C_16_H_26_N_4_^4+^·2.5ClO_4_^−^·1.5NO_3_^−^	*F*(000) = 638
*M**_r_* = 616.05	*D*_x_ = 1.697 Mg m^−^^3^
Monoclinic, *P*2_1_/*n*	Mo *K*α radiation, λ = 0.71073 Å
Hall symbol: -P 2yn	Cell parameters from 73 reflections
*a* = 8.427 (3) Å	θ = 5.0–12.4°
*b* = 12.637 (3) Å	µ = 0.41 mm^−^^1^
*c* = 11.834 (3) Å	*T* = 298 K
β = 106.97 (2)°	Irregular, pale yellow
*V* = 1205.4 (6) Å^3^	0.6 × 0.4 × 0.4 mm
*Z* = 2	

### Data collection

**Table:**

Siemens P4 diffractometer	*R*_int_ = 0.057
Radiation source: fine-focus sealed tube	θ_max_ = 25.1°, θ_min_ = 2.4°
graphite	*h* = −10→10
ω scans	*k* = −15→15
6392 measured reflections	*l* = −14→14
2125 independent reflections	3 standard reflections every 97 reflections
1757 reflections with *I* > 2σ(*I*)	intensity decay: <1%

### Refinement

**Table:**

Refinement on *F*^2^	Primary atom site location: structure-invariant direct methods
Least-squares matrix: full	Secondary atom site location: difference Fourier map
*R*[*F*^2^ > 2σ(*F*^2^)] = 0.050	Hydrogen site location: inferred from neighbouring sites
*wR*(*F*^2^) = 0.119	H-atom parameters constrained
*S* = 1.14	*w* = 1/[σ^2^(*F*_o_^2^) + (0.0221*P*)^2^ + 1.6735*P*] where *P* = (*F*_o_^2^ + 2*F*_c_^2^)/3
2125 reflections	(Δ/σ)_max_ < 0.001
218 parameters	Δρ_max_ = 0.25 e Å^−^^3^
8 restraints	Δρ_min_ = −0.27 e Å^−^^3^
0 constraints	

### Fractional atomic coordinates and isotropic or equivalent isotropic displacement parameters (Å^2^)

**Table:**

	*x*	*y*	*z*	*U*_iso_*/*U*_eq_	Occ. (<1)
N1	−0.1084 (4)	0.6532 (2)	0.2742 (2)	0.0430 (7)	
H1A	−0.2090	0.6242	0.2538	0.065*	
H1B	−0.1163	0.7215	0.2897	0.065*	
H1C	−0.0445	0.6208	0.3381	0.065*	
C2	−0.0347 (4)	0.6416 (2)	0.1763 (3)	0.0326 (7)	
C3	−0.1440 (4)	0.6359 (2)	0.0652 (3)	0.0399 (8)	
H3A	−0.2578	0.6371	0.0546	0.048*	
C4	−0.0826 (5)	0.6283 (2)	−0.0310 (3)	0.0434 (8)	
H4A	−0.1553	0.6235	−0.1070	0.052*	
C5	0.0852 (5)	0.6280 (3)	−0.0143 (3)	0.0430 (8)	
H5A	0.1263	0.6245	−0.0791	0.052*	
C6	0.1929 (4)	0.6327 (2)	0.0979 (3)	0.0394 (8)	
H6A	0.3066	0.6313	0.1082	0.047*	
C7	0.1348 (4)	0.6396 (2)	0.1969 (3)	0.0311 (7)	
C8	0.2604 (4)	0.6487 (2)	0.3159 (3)	0.0371 (7)	
H8A	0.2095	0.6834	0.3696	0.044*	
H8B	0.3513	0.6928	0.3091	0.044*	
N9	0.3283 (3)	0.5437 (2)	0.3671 (2)	0.0350 (6)	
H9A	0.2457	0.5053	0.3806	0.042*	
H9B	0.3660	0.5085	0.3140	0.042*	
C10	0.4644 (4)	0.5534 (3)	0.4787 (3)	0.0380 (8)	
H10A	0.4225	0.5862	0.5384	0.046*	
H10B	0.5509	0.5985	0.4662	0.046*	
Cl1	−0.00237 (10)	0.35712 (6)	0.40133 (7)	0.0396 (2)	
O1	−0.1221 (4)	0.3829 (3)	0.4604 (3)	0.0752 (9)	
O2	0.0116 (4)	0.4419 (2)	0.3253 (2)	0.0643 (8)	
O3	0.1540 (4)	0.3438 (2)	0.4887 (2)	0.0648 (8)	
O4	−0.0495 (4)	0.2635 (2)	0.3332 (3)	0.0647 (8)	
Cl2	0.5512 (9)	0.4371 (6)	0.1804 (8)	0.0358 (15)	0.25
O5	0.587 (4)	0.5336 (15)	0.244 (2)	0.039 (7)	0.25
O6	0.5011 (19)	0.4701 (12)	0.0603 (8)	0.083 (4)	0.25
O7	0.415 (4)	0.394 (4)	0.211 (4)	0.083 (14)	0.25
O8	0.675 (2)	0.3628 (14)	0.186 (2)	0.052 (5)	0.25
N11	0.5525 (14)	0.4327 (9)	0.2076 (9)	0.068 (4)	0.75
O12	0.5925 (19)	0.5250 (7)	0.2380 (11)	0.071 (4)	0.75
O13	0.4469 (14)	0.3860 (9)	0.2416 (13)	0.056 (2)	0.75
O14	0.6277 (12)	0.3811 (8)	0.1526 (9)	0.099 (3)	0.75

### Atomic displacement parameters (Å^2^)

**Table:**

	*U*^11^	*U*^22^	*U*^33^	*U*^12^	*U*^13^	*U*^23^
N1	0.0417 (16)	0.0511 (17)	0.0434 (15)	0.0065 (14)	0.0236 (13)	0.0043 (13)
C2	0.0406 (18)	0.0254 (14)	0.0364 (16)	0.0035 (13)	0.0186 (14)	0.0037 (13)
C3	0.0373 (18)	0.0331 (16)	0.0472 (19)	0.0030 (14)	0.0089 (15)	0.0045 (15)
C4	0.062 (2)	0.0306 (16)	0.0344 (17)	0.0060 (16)	0.0086 (16)	0.0029 (14)
C5	0.065 (3)	0.0361 (18)	0.0336 (17)	0.0020 (17)	0.0231 (17)	0.0038 (14)
C6	0.0451 (19)	0.0333 (16)	0.0475 (18)	−0.0014 (15)	0.0255 (16)	0.0056 (15)
C7	0.0393 (18)	0.0221 (14)	0.0347 (15)	−0.0016 (13)	0.0153 (13)	0.0033 (12)
C8	0.0392 (18)	0.0305 (16)	0.0421 (17)	−0.0037 (14)	0.0128 (15)	−0.0009 (14)
N9	0.0333 (15)	0.0368 (14)	0.0337 (13)	−0.0012 (11)	0.0081 (12)	−0.0022 (11)
C10	0.0333 (18)	0.0418 (18)	0.0355 (17)	−0.0007 (14)	0.0047 (14)	−0.0050 (14)
Cl1	0.0461 (5)	0.0334 (4)	0.0467 (5)	−0.0010 (4)	0.0250 (4)	0.0020 (3)
O1	0.069 (2)	0.088 (2)	0.089 (2)	0.0045 (17)	0.0545 (18)	−0.0055 (18)
O2	0.084 (2)	0.0519 (16)	0.0603 (16)	−0.0161 (15)	0.0263 (15)	0.0158 (13)
O3	0.0577 (18)	0.0691 (18)	0.0606 (16)	0.0068 (15)	0.0065 (14)	−0.0013 (14)
O4	0.0669 (19)	0.0407 (14)	0.086 (2)	−0.0074 (13)	0.0213 (16)	−0.0149 (14)
Cl2	0.028 (3)	0.033 (3)	0.051 (3)	−0.006 (2)	0.018 (2)	−0.007 (2)
O5	0.039 (13)	0.049 (16)	0.039 (9)	0.007 (10)	0.026 (9)	0.002 (9)
O6	0.105 (11)	0.110 (10)	0.037 (6)	−0.004 (9)	0.027 (7)	0.017 (7)
O7	0.059 (14)	0.12 (2)	0.08 (3)	−0.036 (15)	0.030 (17)	−0.020 (16)
O8	0.029 (7)	0.032 (6)	0.094 (12)	0.010 (6)	0.019 (7)	−0.005 (7)
N11	0.070 (6)	0.079 (7)	0.060 (6)	0.024 (5)	0.026 (4)	−0.002 (4)
O12	0.082 (8)	0.029 (4)	0.118 (7)	−0.005 (4)	0.055 (6)	−0.006 (4)
O13	0.060 (5)	0.052 (3)	0.062 (6)	−0.016 (3)	0.028 (5)	−0.003 (3)
O14	0.104 (7)	0.097 (5)	0.122 (8)	0.031 (4)	0.075 (6)	−0.015 (5)

### Geometric parameters (Å, °)

**Table:**

N1—C2	1.473 (4)	N9—C10	1.480 (4)
N1—H1A	0.8900	N9—H9A	0.9000
N1—H1B	0.8900	N9—H9B	0.9000
N1—H1C	0.8900	C10—C10^i^	1.503 (6)
C2—C3	1.369 (4)	C10—H10A	0.9700
C2—C7	1.378 (4)	C10—H10B	0.9700
C3—C4	1.385 (5)	Cl1—O4	1.421 (3)
C3—H3A	0.9300	Cl1—O1	1.423 (3)
C4—C5	1.369 (5)	Cl1—O2	1.426 (3)
C4—H4A	0.9300	Cl1—O3	1.428 (3)
C5—C6	1.374 (5)	Cl2—O8	1.389 (9)
C5—H5A	0.9300	Cl2—O7	1.409 (10)
C6—C7	1.398 (4)	Cl2—O5	1.417 (10)
C6—H6A	0.9300	Cl2—O6	1.422 (9)
C7—C8	1.498 (4)	N11—O14	1.222 (8)
C8—N9	1.501 (4)	N11—O13	1.229 (8)
C8—H8A	0.9700	N11—O12	1.238 (8)
C8—H8B	0.9700		
			
C2—N1—H1A	109.5	H8A—C8—H8B	107.8
C2—N1—H1B	109.5	C10—N9—C8	113.1 (2)
H1A—N1—H1B	109.5	C10—N9—H9A	109.0
C2—N1—H1C	109.5	C8—N9—H9A	109.0
H1A—N1—H1C	109.5	C10—N9—H9B	109.0
H1B—N1—H1C	109.5	C8—N9—H9B	109.0
C3—C2—C7	122.8 (3)	H9A—N9—H9B	107.8
C3—C2—N1	116.1 (3)	N9—C10—C10^i^	110.7 (3)
C7—C2—N1	121.0 (3)	N9—C10—H10A	109.5
C2—C3—C4	119.0 (3)	C10^i^—C10—H10A	109.5
C2—C3—H3A	120.5	N9—C10—H10B	109.5
C4—C3—H3A	120.5	C10^i^—C10—H10B	109.5
C5—C4—C3	120.0 (3)	H10A—C10—H10B	108.1
C5—C4—H4A	120.0	O4—Cl1—O1	110.36 (19)
C3—C4—H4A	120.0	O4—Cl1—O2	109.21 (17)
C4—C5—C6	120.2 (3)	O1—Cl1—O2	109.82 (19)
C4—C5—H5A	119.9	O4—Cl1—O3	111.00 (17)
C6—C5—H5A	119.9	O1—Cl1—O3	107.96 (19)
C5—C6—C7	121.2 (3)	O2—Cl1—O3	108.46 (19)
C5—C6—H6A	119.4	O8—Cl2—O7	112 (2)
C7—C6—H6A	119.4	O8—Cl2—O5	121.0 (17)
C2—C7—C6	116.8 (3)	O7—Cl2—O5	105 (3)
C2—C7—C8	125.2 (3)	O8—Cl2—O6	104.3 (13)
C6—C7—C8	117.9 (3)	O7—Cl2—O6	110 (2)
C7—C8—N9	113.2 (2)	O5—Cl2—O6	103.4 (13)
C7—C8—H8A	108.9	O14—N11—O13	117.1 (12)
N9—C8—H8A	108.9	O14—N11—O12	121.2 (12)
C7—C8—H8B	108.9	O13—N11—O12	121.3 (12)
N9—C8—H8B	108.9		
			
C7—C2—C3—C4	−0.3 (5)	C5—C6—C7—C2	−0.1 (4)
N1—C2—C3—C4	178.0 (3)	C5—C6—C7—C8	−177.7 (3)
C2—C3—C4—C5	−0.8 (5)	C2—C7—C8—N9	98.7 (3)
C3—C4—C5—C6	1.5 (5)	C6—C7—C8—N9	−83.9 (3)
C4—C5—C6—C7	−1.0 (5)	C7—C8—N9—C10	174.1 (3)
C3—C2—C7—C6	0.7 (4)	C8—N9—C10—C10^i^	−175.9 (3)
N1—C2—C7—C6	−177.5 (3)	O8—Cl2—O6—O6^ii^	−79 (5)
C3—C2—C7—C8	178.2 (3)	O7—Cl2—O6—O6^ii^	160 (6)
N1—C2—C7—C8	−0.1 (4)	O5—Cl2—O6—O6^ii^	48 (6)

Symmetry codes: (i) −*x*+1, −*y*+1, −*z*+1; (ii) −*x*+1, −*y*+1, −*z*.

### Hydrogen-bond geometry (Å, °)

**Table:**

*D*—H···*A*	*D*—H	H···*A*	*D*···*A*	*D*—H···*A*
N1—H1C···O2	0.89	2.32	2.856 (4)	118
N1—H1B···O13^iii^	0.89	2.61	3.270 (13)	132
N9—H9B···O5	0.90	2.27	2.96 (3)	133
N9—H9B···O12	0.90	2.35	3.055 (15)	136
N9—H9A···O2	0.90	2.05	2.872 (4)	151
N9—H9A···O3	0.90	2.64	3.441 (4)	149
N9—H9B···O7	0.90	2.02	2.89 (5)	163
N9—H9B···O13	0.90	1.98	2.836 (13)	157
N1—H1A···O5^iv^	0.89	2.04	2.91 (3)	165
N1—H1A···O12^iv^	0.89	2.05	2.920 (13)	164
N1—H1B···O8^iii^	0.89	1.90	2.78 (2)	170
N1—H1B···O14^iii^	0.89	2.14	3.026 (11)	174
N1—H1C···O1^v^	0.89	2.39	3.207 (4)	153

Symmetry codes: (iii) −*x*+1/2, *y*+1/2, −*z*+1/2; (iv) *x*−1, *y*, *z*; (v) −*x*, −*y*+1, −*z*+1.

## References

[bb1] Carta, D., Cao, G. & D’Angeli, C. (2003). *Environ. Sci. Pollut. Res. Int* **10**, 390–394. 10.1065/espr2001.12.104.8 14699998

[bb2] Macrae, C. F., Edgington, P. R., McCabe, P., Pidcock, E., Shields, G. P., Taylor, R., Towler, M. & van de Streek, J. (2006). *J. Appl. Cryst.* **39**, 453–457.

[bb3] Parra, J. B., Ania, C. O., Arenillas, A., Rubiera, F. & Pis, J. J. (2004). *Appl. Surf. Sci* **238**, 304–308.

[bb4] Pohorely, M., Vosecky, M., Hejdova, P., Puncochar, M., Skoblja, S., Staf, M., Vosta, J., Koutsky, B. & Svoboda, K. (2006). *Fuel*, **85**, 2458–2468.

[bb5] Rodríguez de Barbarín, C., Bernès, S., Nájera, B., Elizondo, P. & Cerda, P. (2007). *Acta Cryst.* E**63**, o549–o550. 10.1107/S010827010704156X 17917232

[bb6] Sheldrick, G. M. (2008). *Acta Cryst.* A**64**, 112–122. 10.1107/S0108767307043930 18156677

[bb7] Siemens (1996). *XSCANS*. Siemens Analytical X-ray Instruments Inc., Madison, Wisconsin, USA.

